# The Plague

**Published:** 1897-02-20

**Authors:** 


### The Plague.

The literature on the subject of plague tends to become voluminous, and it seems desirable to set before our readers some of the considerations which will have to be borne in mind in any attempt to frame useful measures for its treatment, whether from a public or an individual point of view. It seems generally admitted that, given the old conditions, plague might spread now as it so often did between the fourteenth century and the seventeenth. In considering how to control an admittedly infective disease, the first thing is to ascertain by what route the infection leaves the sufferer that has been; the next, in what way it gains access to the sufferer that is to be. Now, it seems quite certain that the infection of plague is not given off by the breath, or exhaled from the body of the patient. For practical purposes we must accept the teaching of experience, and what was learned at Hong Kong, even if we had no other knowledge pointing in the same direction, all goes to show that a "plague atmosphere," so far as that means an atmosphere befouled by the bodily exhalations of plague patients, is not capable of spreading the infection. As Dr. Lowson says, if infection were conveyed by the breath more deaths would certainly have occurred among those engaged in plague work. The party employed in house-to-house visitation spent two hours twice a day entering the most infective houses in the city, yet no one took the disease. During the early days of the epidemic, also, those on board the hospital-ship were frequently fourteen hours on duty, and the atmosphere was so bad from the smell of the patients that vomiting was often induced, yet none of the attendants who remained on duty were attacked. In the Chinese hospital, again, not one of the Chinese attendants got plague, although they were breathing as thick a "plague atmosphere" as one could desire. The soldiers, however, who volunteered for cleansing duty, those who had to remove the dirt after stirring it up, got plague; but that only happened before they began to disinfect the ground before disturbing it. After that was done none of them caught the disease. This gives the clue to the mode in which the infection is usually conveyed, viz., by dirt and dust containing the specific infection.

How then does the dirt, the soil, the dust of the house become infected? To decide this we have to inquire how the infection leaves the body, and it is here that bacteriology is of service. The infective bacilli are present in the discharge from the buboes when the patient lives long enough for these to mature and suppurate. This, however, must be a comparatively subordinate mode of spread, for in the midst of an active epidemic the majority die before discharge occurs. The bacilli are also contained in the sputum and in the fur on the tongue. The milk would not seem to be infectious, as Dr. Lowson says that in the outbreak at Hong Kong many children who were suckled by their plague-stricken mothers lived.

So far all culture experiments with the sweat have failed to show the presence of the bacilli therein. The fæces, however, are infectious. In every case investigated by Staff-Surgeon Wilm, at Hong Kong, the plague bacillus was found in them, and there can be very little doubt that the reason why plague has so often been found to affect especially, and sometimes exclusively, the lower-class natives, is that among them there is so much carelessness in the disposal of the excreta. Soil becomes infected and then the dust is breathed, or swallowed, or by direct inoculation of scratches the disease is introduced.

So much for facts. The lessons are fairly plain to those who care to read. We hear much of medical examination, and separation of the infected from the healthy. But these things will not stop plague. Animals suffer along with man, and will spread the infection so long as it is allowed to escape in an active form. What is required is not so much the segregation of the patient as the destruction of the infective particles which come from that patient during the progress of the disease. By the free use of antiseptics to all wounds and the cremation of all dressings; by the careful collection of the excreta and their cremation or disinfection; by the cremation of the bodies of the dead; by the use of powerful disinfectants for the cleansing of all vessels and for the hands of the attendants, as is done in cholera, but with even more minute attention to details; and by the complete disinfection of every house in which the disease has appeared, we may hope to stop the spread even of plague.

In regard to our own country, it will be possible to speak with some confidence if once it is recognised by the sanitary authorities that the best point at which to intercept the infection is as it leaves the body. In India the matter is different. The customs of the country, the condition of filth in the midst of which the majority of the people live, as shown by the readiness with which cholera spreads among them, and their unwillingness to report the cases, make us very doubtful of the issue. Plague will die out there, as it has died out in many places before; but here, if set about scientifically, we can have no doubt that the disease—if it should appear—could be almost as readily controlled as cholera.

